# Supplementary material for: Experimental Infection of Mid-Gestation Pregnant Female and Intact Male Sheep with Zika Virus
Source: Viruses. 2020 Mar 7;12(3):291. doi: 10.3390/v12030291 (PMC7150993; doi:10.3390/v12030291)
Supplement: Supplementary file 1 [file viruses-12-00291-s001.pdf]

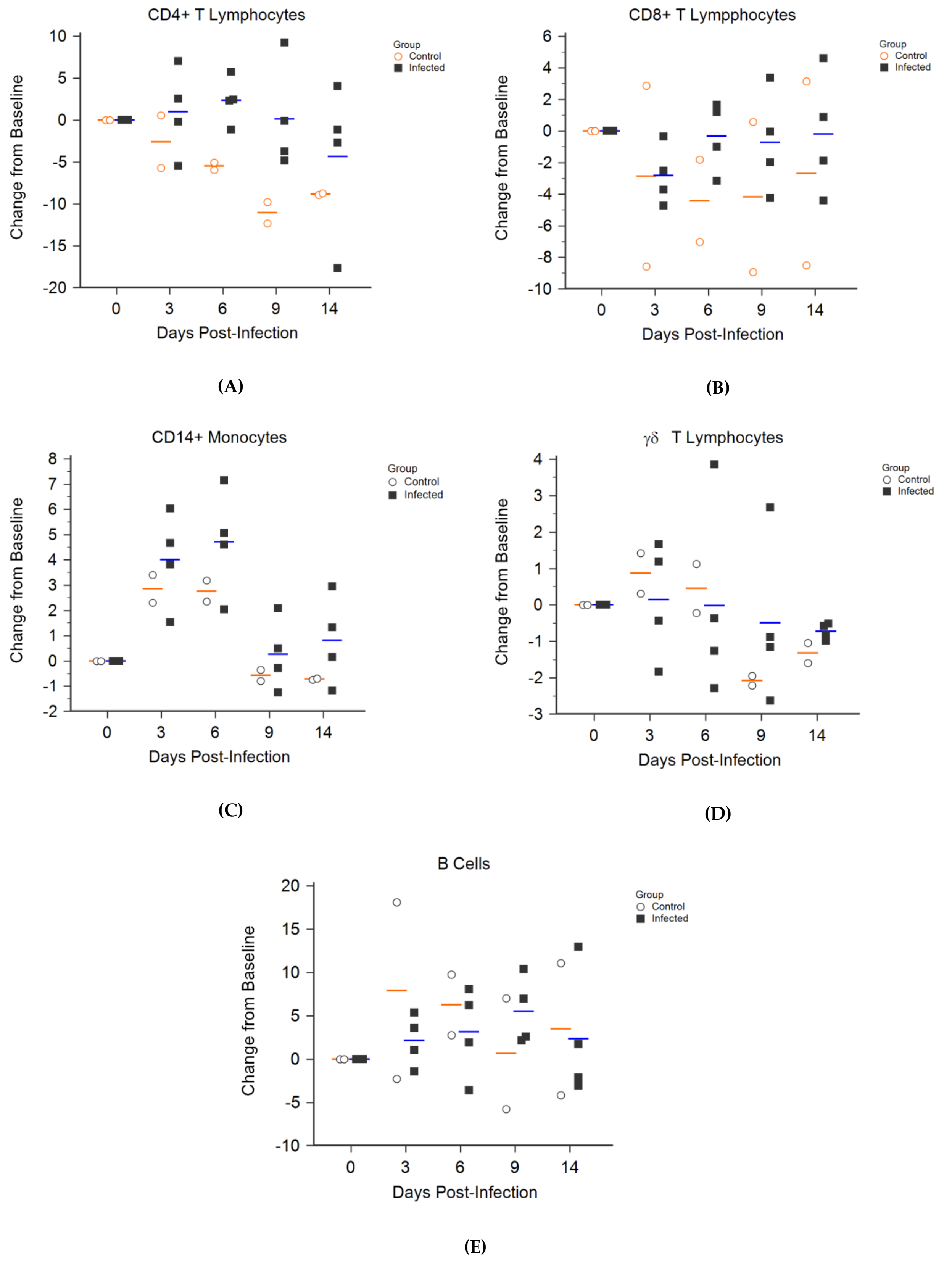

Figure S1. PBMC phenotypes measured by FACS over the course of the study period, reported as the change in percentage of cells from initial pre-infection measurement (standardized to 0 on the y-axis); horizontal lines indicate the mean change in percentage for each group, where blue represents infected animals and orange represents control animals. Specific phenotypes measured included: A) CD4+ T lymphocytes; B) CD8+ T lymphocytes; C) CD14+ monocytes; D) γδ T lymphocytes; and E) B cells.
